# Unraveling the cocoon: A case report on preoperative ambiguity and definitive surgical management of encapsulating peritoneal sclerosis

**DOI:** 10.1016/j.ijscr.2025.112065

**Published:** 2025-10-14

**Authors:** Suman Khadka, Diwakar Koirala, Ramesh Sapkota, Tek Nath Yogi, Kriti Basnet, Vijay Shrestha

**Affiliations:** a Department of Surgery, B.P. Koirala Institute of Health Sciences, Dharan, Nepal; b District Hospital Ilam, Nepal

**Keywords:** Abdominal cocoon, Sclerosing peritonitis, Intestinal obstruction, Adhesiolysis, Laparotomy, Case report

## Abstract

**Introduction:**

Abdominal cocoon, or sclerosing encapsulating peritonitis (SEP), is a rare condition characterized by a fibrocollagenous membrane encasing the intestines, often leading to obstruction. First described in 1907, its etiology remains unclear, with primary (idiopathic) and secondary forms linked to prior surgeries, infections, or systemic diseases. Preoperative diagnosis remains challenging due to nonspecific symptoms.

**Case presentation:**

A 49-year-old female presented with abdominal pain, vomiting, and constipation. Imaging revealed dilated bowel loops and adhesions. Exploratory laparotomy confirmed SEP, with histopathology showing chronic active colitis and fibrous peritoneum. Adhesiolysis and loop ileostomy were performed, with the patient discharged in stable condition.

**Discussion:**

SEP is classified into three anatomical types, with idiopathic cases often lacking identifiable risk factors. Diagnostic imaging (CT/MRI) aids suspicion, but definitive diagnosis typically requires laparotomy. The condition must be differentiated from peritoneal encapsulation, a distinct entity. Management depends on severity, ranging from membrane excision to bowel resection. A review of 14 published cases highlights laparotomy as the gold standard for both diagnosis and treatment, though laparoscopic approaches are emerging.

**Conclusion:**

SEP remains a diagnostic dilemma, necessitating high clinical suspicion in cases of unexplained obstruction. Surgical intervention is often curative, but meticulous technique is vital to avoid complications. Further research into biomarkers and minimally invasive diagnostic tools is warranted.

## Highlights

• CT imaging aids preoperative suspicion but diagnosis requires laparotomy.
• A cocoon abdomen presents with bowel obstruction and nonspecific symptoms.
• Membrane excision and adhesiolysis remain the mainstay of surgical treatment.
• Histopathology confirms fibrous encapsulation with chronic active colitis.
• Early intervention prevents complications like bowel necrosis and sepsis.

## 1 Introduction

A cocoon is a dense protective covering that envelops a silkworm or similar insect during its transition from larva to pupa. Abdominal cocoon, also known as sclerosing encapsulating peritonitis, is a rare condition that predominantly affects women [1] and is extremely rare in men. In this condition, a thick peritoneal membrane envelops the intestines, causing the bowel loops to adhere to one another. A varying length of the bowel may be involved [2]. Because of the lack of distinct clinical signs or symptoms, diagnosing cocoon abdomen before surgery is quite challenging [3]. In 1907, Owtschinnikow was the first to describe the encapsulation of the intestines by a fibrocollagenous membrane, introducing the term *peritonitis chronica fibrosa incapsulata* [4]. Today, the condition is commonly known as Encapsulating Peritoneal Sclerosis, Abdominal Cocoon, or Sclerosing Encapsulating Peritonitis [5]. The exact cause of this condition remains uncertain. It can be classified as either primary (idiopathic) or secondary, the latter often linked to factors such as prior abdominal surgeries, complicated abdominal wall hernias, renal or liver transplants, and peritoneo-venous shunts in patients with liver cirrhosis [6]. Most patients present with abdominal pain and intestinal obstruction which is usually diagnosed intraoperatively. CT-scan or barium study can help in suspecting the condition before surgery [7].

## 2 Case presentation

A 49-year-old female was admitted to a tertiary care centre with a primary complaint of abdominal pain for 7 days, vomiting for 5 days and constipation for 4 days. There was no reported history of dysuria or urinary frequency. The patient had no significant past medical or family history, followed a mixed diet, was a former smoker, and had no known drug allergies or prior adverse drug reactions.

Clinical assessment indicated a conscious, oriented, and afebrile patient with stable vital signs. Abdominal examination revealed distension with an inverted umbilicus. Laboratory results included sodium (136 mmol/L), potassium (4.0 mmol/L), creatinine (0.9 mg/dL), urea (25 mg/dL), and a prothrombin time of 12.5 s.

**Fig. 1:**
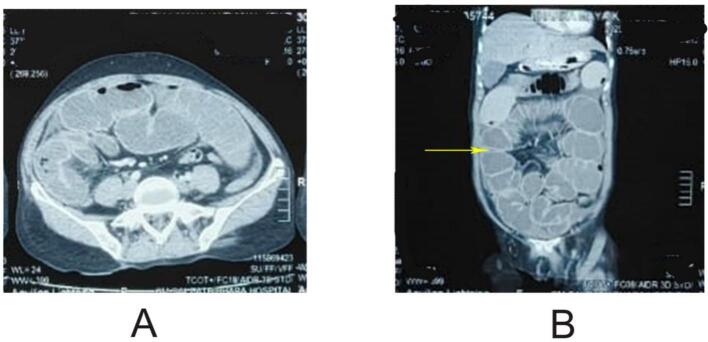
A: transverse slice B: coronal slice showing dilated loops and adhesions.

USG abdomen and Pelvis revealed several dilated central and peripheral bowel loops with interbowel loop collection. CT scan abdomen and pelvis showed small dilated bowel loops of maximum caliber about 4.5 cm and air fluid level within. A short segment (approximately 10 cm) of mildly thickened and enhancing terminal ileum was noted in the right iliac fossa region, as shown in Fig. 1.

**Fig. 2:**
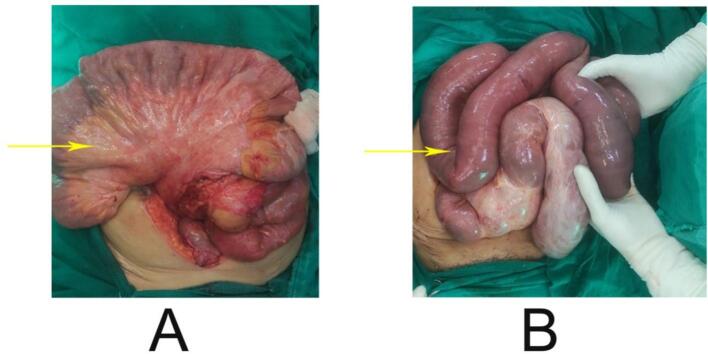
A: Yellow arrow shows thickened mesentery, B: yellow arrow shows dilated bowel loops.

After all the relevant investigations including TB work up which was negative exploratory Laparotomy with adhesiolysis with loop ileostomy under GA was performed. Histopathological examination demonstrated chronic active colitis with mild inflammatory infiltrate in the lamina propria, absence of granulomas or malignancy, and no evidence of microbial infection on special staining, suggestive of fibrous peritoneum confirming the diagnosis of a cocoon abdomen as shown in Fig. 2.

Following a seven-day hospitalization, the patient was discharged in stable condition with prescribed medications, including an antibiotic, proton pump inhibitor, thyroid supplement, multivitamin, and as-needed antispasmodic syrup. The patient was advised to follow up after ten days with relevant reports, monitor intake and output, and consult a surgeon if any danger signs occurred (Table 1).

**Table 1:** Clinical timeline.

Days	Event
Day 0	Onset of abdominal pain
Day 2	Vomiting began
Day 3	Constipation developed
Day 7	Hospital admission, investigations performed
Day 8	Exploratory laparotomy with adhesiolysis and loop ileostomy
Day 14	Discharged in stable condition

## 3 Discussion

Abdominal cocoon syndrome was initially identified over a century ago and was originally designated as *peritonitis chronica fibrosa incapsulata*, a term used to describe the fibrocollagenous membrane encapsulating the intestines [4]. In the majority of cases, the etiology of sclerosing peritonitis remains unidentified. Abdominal cocoon syndrome must be distinguished from peritoneal encapsulation, a distinct entity first described in 1868, as they differ in both etiology and management. In peritoneal encapsulation, the small intestine is enclosed posteriorly by an accessory yet anatomically normal peritoneal membrane, which is adherent to the ascending, descending, and transverse segments of the colon [8]. Peritoneal encapsulation has normal peritoneal layer however, abdominal cocoon will always have inflamed peritoneum secondary to some inflammatory lesions.

Sclerosing encapsulating peritonitis (SEP) is further classified into primary (idiopathic) and secondary forms based on its underlying etiology [9]. The primary form, also known as abdominal cocoon disease, is believed to result from developmental anomalies such as aberrant embryonic body folding, defective mesodermal differentiation, and dysplasia of the dorsal mesentery of the intestine. It is frequently associated with congenital anatomical abnormalities, including absence of the omentum or gastrocolic ligament, visceral transposition, intestinal or colonic malrotation, cryptorchidism, hernias, and other related anomalies [10]. The secondary form of sclerosing encapsulating peritonitis may develop as a result of various predisposing conditions, including chronic peritoneal inflammation with fibroblastic proliferation, systemic lupus erythematosus, liver cirrhosis, endometriotic cysts, prior abdominal trauma or surgical interventions, abdominal tuberculosis, and intra-abdominal malignancies [11].

Abdominal cocoon is classified into three types based on the extent of membrane encapsulation [12]:

• Type I: Partial encapsulation of the small intestine by the fibrous membrane.
• Type II: Complete encapsulation of the entire small intestine.
• Type III: Encapsulation of the entire small intestine along with other intra-abdominal organs, such as the appendix, cecum, ascending colon, and ovaries.

In numerous instances of idiopathic encapsulating peritoneal sclerosis (EPS), no identifiable association with known risk factors can be established [13]. Two key considerations have been proposed in the pathogenesis of EPS. Firstly, these forms often involve peritoneal abnormalities in conjunction with systemic connective tissue disorders, particularly affecting serosal membranes, as observed in cases of abdominal trauma. This supports the hypothesis that immunopathogenic mechanisms play a significant role in EPS development. Secondly, a potential genetic predisposition is suggested by the higher incidence of EPS among women from subtropical regions and the presence of familial cases, such as multifocal fibrosclerosis [14].

The nonspecific nature of early clinical manifestations makes definitive preoperative diagnosis of encapsulating peritoneal sclerosis (EPS) particularly challenging [15]. Abdominal radiographs generally demonstrate low diagnostic sensitivity, most often showing dilated small intestinal loops, as observed in our patient. Although not present in this case, peritoneal and bowel wall calcifications have been reported. Ultrasonography revealed dilated peripheral loops, consistent with previous descriptions; in other reports, it has also demonstrated ascitic fluid with or without loculated collections and a membranous structure encasing the bowel. Additionally, clustering of bowel loops with adhesions may produce a mass-like appearance, though this feature was absent in our patient [16].

Currently, no specific serological biomarkers have been identified for the diagnosis of EPS. Abdominal computed tomography (CT) demonstrates a higher sensitivity, ranging from 70 % to 90 %, in identifying the underlying cause of high-grade small bowel obstruction; however, its use should be judicious, particularly in younger patients, due to the associated radiation exposure risks. Magnetic resonance imaging (MRI) has recently emerged as an additional valuable imaging modality in the diagnosis of encapsulating peritoneal sclerosis (EPS).

Several reports have been described regarding the cases. Each individual case is in the tabulated form below for better comparison in Table 2.

**Table 2:** Showing complaints and treatment modalities.

S. no.	Authors	Patient complaints	Interventions
1.	Zhou H et al. [17]	A 58 year old male with paroxysmal colicky abdominal pain with abdominal distension	Exploratory laparotomy with sac release
2.	Miyagishima et al. [18]	A 64 year old female with abdominal distension for 2 months with history of oopherectomy for ovarian cyst	Adhesiolysis
3.	Edmundson et al. [19]	A 66 year old man with nausea, vomiting and ascites	Laparotomy with meticulous dissection of the membrane
4.	Karl AP et al. [20]	A 37 year old man with abdominal pain and weight loss over a period of few months with tuberculosis positive in PCR	Exploratory laparotomy with adhesiolysis and resection followed by quadruple tuberculostatic treatment
5.	Hassan et al. [1]	Case 1: A 30 year old male with colicky abdominal pain for 12 h with 2 episodes of bilious vomiting	Case 1: Release of bowel loops by adhesiolysis of the membrane
			Case 2: Release of adhesions
		Case 2: A 35 year old male with colicky abdominal pain in right lower quadrant	
6.	Deutsch GB et al. [21]	An 85 year old man with history of nausea and vomiting with occupational exposure to asbestos	Exploratory laparotomy with adhesiolysis with resection and primary anastomosis
7.	Singh D et al. [22]	A 31 year old male with colicky central abdominal pain and some episodes of vomiting	Exploratory laparotomy with adhesiolysis of the membrane
8.	Saqib SU et al. [23]	A 35 year old male with one day history of generalized abdominal pain, vomiting and constipation in grade III shock with metabolic acidosis	Exploratory laparotomy with extensive adhesiolysis with resection of the membrane
9.	Rehman et al. [12]	A 31 year old male with generalized abdominal pain and distension for 10 days with PCR Tuberculosis positive	Exploratory laparotomy with adhesiolysis followed by antitubercular medication
10.	Bourabaa S et al. [24]	An 87 year old female with prior history of abdominal surgery with history of abdominal pain, distension and vomiting for 4 days	Exploratory laparotomy with adhesiolysis and meticulous excision.
11.	Kaur S et al. [25]	A 41 year old male with vomiting and loss of weight since 2 months	Exploratory laparotomy with adhesiolysis
12.	Aziz et al. [26]	A 29 year old male with abdominal pain, vomiting and constipation since few days	Laparoscopic adhesiolysis and laparoscopic adhesiolysis
13.	Chen et al. [27]	A 50 year old man with intermittent abdominal pain for several days with history of CAPD since 17 years for ESRD	Exploratory laparotomy with intra-abdominal abscess drainage
14.	Mishra et al. [28]	A 32 year old male with constipation, vomiting and abdominal pain for 4 days	Exploratory laparotomy with peritoneal lavage with excision of the gangrenous bowel.

The data presented in the table confirm that, although radiographic imaging can assist in the diagnostic process, definitive diagnosis and management are typically achieved only through Laparotomy, underscoring the challenges associated with preoperative identification of the condition.

The following 5 points of suggestion have been made for the management of abdominal cocoon after study of 32 patients [29].

1. Complete Membrane Excision: If the encapsulating membrane is easily separable, it should be removed as thoroughly as possible.
2. Intestinal Resection: In cases of localized bowel necrosis or perforation, resection of the affected segment is mandatory.
3. Caution During Enterolysis: When enterolysis is technically difficult, extreme care must be taken to avoid accidental injury to the intestine.
4. Management of Intraoperative Injuries: If an intestinal wound occurs during surgery, the most proximal site should be exteriorized as a temporary enterostomy. Temporary enterostomy is also strongly recommended following bowel resection.
5. Non-operative Approach in Select Cases: When SEP is not associated with acute intestinal obstruction—such as incidental findings, minimally symptomatic cases, or subacute obstruction with ascites—routine surgical or preoperative intervention is generally not required.

In this case, a loop ileostomy was chosen in addition to adhesiolysis due to concerns of bowel edema, compromised peristalsis, and risk of anastomotic leak in the immediate postoperative period. Temporary fecal diversion allowed decompression, reduced intraluminal pressure, and provided a safer recovery window.

## 4 Conclusion

Sclerosing encapsulating peritonitis (SEP), or abdominal cocoon, is a rare but important cause of intestinal obstruction, usually diagnosed intraoperatively due to nonspecific clinical and radiological findings. This case highlights the diagnostic challenges and underscores the value of CT in raising suspicion, while confirming laparotomy as the definitive approach. Management should be tailored to disease severity, with adhesiolysis as the mainstay and bowel resection reserved for nonviable segments; temporary enterostomy may be warranted in selected cases. Literature supports laparotomy as the gold standard, though minimally invasive techniques show emerging potential. Future research should prioritize biomarkers and advanced imaging to facilitate earlier diagnosis. A multidisciplinary approach remains essential to optimize outcomes in these complex cases.

The case has been reported in accordance to the SCARE Criteria [30].

## CRediT authorship contribution statement

1. Dr. Suman Khadka, Resident, Department of Surgery, B.P. Koirala Institute of Health Sciences, Dharan, Nepal, Primary Author
2. Dr. Diwakar Koirala, MBBS, Department of Surgery, B.P. Koirala Institute of Health Sciences, Dharan, Nepal, Corresponding Author
3. Dr. Ramesh Sapkota, MBBS, Department of Surgery, B.P. Koirala Institute of Health Sciences, Dharan, Nepal.
4. Dr. Tek Nath Yogi, MBBS, Department of Surgery, B.P. Koirala Institute of Health Sciences, Dharan, Nepal.
5. Dr. Kriti Basnet, Medical Officer, District Hospital Ilam, Nepal
6. Dr. Vijay Shrestha, Associate Professor, Department of Surgery, B.P. Koirala Institute of Health Sciences, Dharan, Nepal.

## Informed consent

Informed consent taken from the patient regarding the reporting of the case.

## Ethical approval

Not required for the case report.

## Guarantor

Dr. Diwakar Koirala is the guarantor of this research.

## Funding

There was no funding involved.

## Declaration of competing interest

The authors of this manuscript declare no conflict of interest.
